# IL-27/Blimp-1 axis regulates the differentiation and function of Tim-3^+^ Tregs during early pregnancy

**DOI:** 10.1172/jci.insight.179233

**Published:** 2024-08-22

**Authors:** Si-Jia Zhao, Xiao-Hui Hu, Xin-Xiu Lin, Yu-Jing Zhang, Jing Wang, Huan Wang, Guang-Shun Gong, Gil Mor, Ai-Hua Liao

**Affiliations:** 1 Institute of Reproductive Health, Center for Reproductive Medicine, Tongji Medical College, Huazhong University of Science and Technology, Wuhan, China.; 2 Department of Obstetrics and Gynecology, First Clinical College Union Hospital, Huazhong University of Science and Technology, Wuhan, China.; 3 C.S. Mott Center for Human Growth and Development, Department of Obstetrics and Gynecology, School of Medicine, Wayne State University, Detroit, Michigan, USA.

**Keywords:** Immunology, Reproductive biology, Obstetrics/gynecology, T cells, Tolerance

## Abstract

Decidual regulatory T cells (Tregs) are essential for successful pregnancy outcome. A subset of Tregs, T cell immunoglobulin and mucin domain-containing protein 3–positive regulatory T cells (Tregs^Tim-3+^), plays a central role in the acceptance of the fetus during early stages of normal pregnancy. The molecular mechanism regulating the differentiation and function of Tregs^Tim-3+^ is unknown. Here, we investigated the role of the transcription factor B lymphocyte-induced maturation protein 1 (Blimp-1) on decidual Treg^Tim-3+^ differentiation. We demonstrated that Blimp-1 enhanced the coexpression of negative costimulatory molecules (Tim-3, T cell immunoreceptor with Ig and ITIM domains, and programmed cell death protein 1) on Tregs and improved their immunosuppressive functions, including increased IL-10 secretion, suppression of effector T cell proliferation, and promotion of macrophage polarization toward the M2 phenotype. Furthermore, we showed that IL-27 regulated the expression of Tim-3 and Blimp-1 through the STAT1 signaling pathway and that transfer of Tregs^Blimp-1+^ into an abortion-prone mouse model effectively reduced embryo absorption rate. We postulated that abnormalities in the IL-27/Blimp-1 axis might be associated with recurrent pregnancy loss (RPL). These findings provided insights for developing more efficient immunotherapies for women with RPL.

## Abstract 2

This was the first study to describe the role of transcription factor B-lymphocyte-induced maturation protein 1 (Blimp-1) in the regulation of decidual Tim-3+Treg cell differentiation and function. Transferring Blimp-1+Tregs could effectively improve pregnancy outcomes in mice.

## Introduction

Decidual regulatory T cells (Tregs) are indispensable for successful embryo implantation and pregnancy outcome and contribute to maternal fetal tolerance at multiple levels (1, 2). Our group initially identified that T cell immunoglobulin and mucin domain-containing protein 3–positive Tregs (Tregs^Tim-3+^) were a unique subset of Tregs, which predominantly accumulated at the decidua of early pregnancy but sparsely existed in the periphery and played an important role in inducing and maintaining normal pregnancy (3). Blocking the Tim-3 signaling pathway in a BALB/c (male) × C57 (female) allogeneic mouse model disrupted the immune tolerance at the materno-fetal interface, ultimately resulting in increased embryo absorption (3). Blocking Tim-3 and cytotoxic T lymphocyte-associated antigen-4 (CTLA-4) suppresses antiinflammatory cytokine production and is detrimental to normal pregnancy (4, 5). Multiple lines of evidence have indicated that Tim-3 signaling is crucial for maintaining the normal pregnancy, and its aberration is linked to recurrent pregnancy loss (RPL) (6, 7). However, the molecular mechanisms underlying the induction of Tim-3 expression and the generation of Tregs^Tim-3+^ in the decidua remain largely unclear.

B lymphocyte-induced maturation protein 1 (Blimp-1), a multifunctional transcription factor encoded by the PR/SET domain 1 (*PRDM1*) gene, plays a crucial role in the differentiation of B cells into plasma cells and the secretion of immunoglobulins (8). Additionally, Blimp-1 determines the cell fate during mammalian embryonic development (9, 10). During mid-to-late embryonic development, Blimp-1 silences the default somatic cell program, allowing a small subset of primordial germ cells to become unresponsive to BMP/Smad signals and acquire a germ cell fate (11). Dysregulation in the Blimp-1 gene disrupts the ability of spiral artery–associated trophoblast giant cells to invade spiral arteries and severely impairs the expansion of the spongy trophoblast layer, leading to the collapse of the labyrinth layer and developmental arrest of the embryo at gestational day 10.5 (12, 13).

Notably, in addition to regulating B cell maturation, Blimp-1 plays a substantial role in regulating Tregs (14, 15). Blimp-1 prevents Foxp3^+^RORγt^+^ Tregs from inducing inflammatory responses by serving as a molecular switch (16). Moreover, the secretion of IL-10 by Foxp3^+^ Tregs is also dependent on Blimp-1 (14). As a downstream target gene of IL-27, Blimp-1, in coordination with the transcription factor c-MAF, regulates the expression of multiple negative costimulatory molecules in CD4^+^ T cells and CD8^+^ T cells, including programmed cell death protein 1 (PD-1), Tim-3, lymphocyte-activation gene 3, and T cell immunoreceptor with Ig and ITIM domains (Tigit) (17, 18). A study on acute myeloid leukemia demonstrated that Blimp-1 could bind to the promoters of PD-1 and Tigit, positively regulate their expression, and impair T cell function with reduced cytokine production and decreased cytotoxicity (19). Additionally, data from the published sequencing databases revealed that Blimp-1 was specifically upregulated in decidual Tregs rather than in peripheral Tregs (20). However, the immunological roles of Blimp-1 at the materno-fetal interface and the regulation of decidual Tregs^Tim-3+^ remain unknown.

These observations raise the question of the degree to which Blimp-1 endows Tregs^Tim-3+^ with immune-regulatory functions and how to utilize this mechanism to improve pregnancy outcomes. Herein, we investigated the dynamic changes of Blimp-1 expression in human decidua during pregnancy and its role on the regulation of Treg differentiation. Interestingly, we observed the highest levels of Blimp-1 expression in the first trimester decidua. Furthermore, we showed that Blimp-1 regulated the expression of negative costimulatory molecules (Tim-3, Tigit, and PD-1) in Tregs, thereby improving their immunosuppressive functions. Transferring Tregs^Blimp-1+^ into an abortion-prone mouse model effectively reduced embryo absorption rate. Taken together, our findings provide deeper insights into the roles of Tregs^Tim-3+^ and its regulator, Blimp-1, in pregnancy maintenance.

## Results

### Expression of Tim-3 is higher in decidual Tregs than in peripheral Tregs.

**Figure 1:**
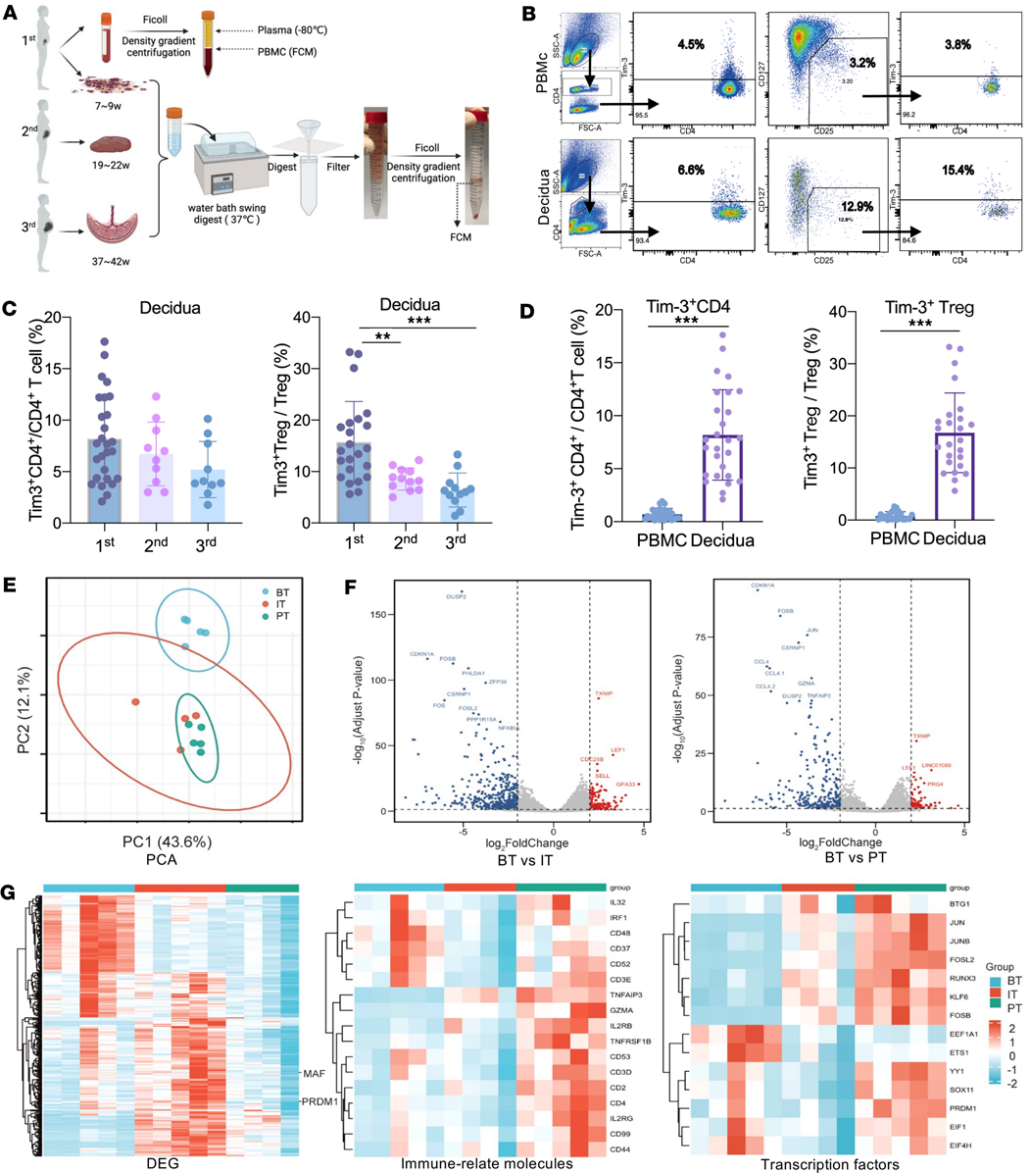
Comparison of the dynamic expression of Tim3 on decidual Tregs and peripheral Tregs during pregnancy. (**A**) Experimental workflow and collection of PBMCs and decidual tissue samples (undergoing digestion in 37°C water bath swing) from early, middle, and late pregnancy for flow cytometry (FCM). (**B**) Gating strategy for peripheral and decidual Tim-3^+^CD4^+^ T cells and Tim-3^+^ Tregs. (**C**) Dynamic changes in the proportion of Tim-3^+^CD4^+^ T cells and Tim-3^+^ Tregs in decidua during the first, second, and third trimesters. (**D**) Comparison of the proportion of Tim-3^+^CD4^+^ T cells and Tim-3^+^ Tregs in peripheral and decidua during the first trimester. (**E**) Principal component analysis (PCA) plot generated of BT, IT, and PT groups by using the R-ggplot2 package. (**F**) Volcano plots showing differentially expressed genes between BT and PT, as well as between BT and IT groups. (**G**) Heatmap displaying all genes, immune-related molecules, and transcription factors in the BT, IT, and PT groups. BT, peripheral Treg; IT, Treg in decidua basalis; PT, Treg in decidua parietalis. Data are represented as the mean ± SEM via 1-way ANOVA test (**C**) and unpaired, 2-tailed Student’s *t* test (**D**). **P < 0.01, ***P < 0.001.

To elucidate Tim-3 expression patterns in Tregs during pregnancy, we collected human decidual samples in the first, second, and third trimesters and peripheral blood in the first trimester (Figure 1A). FCM was used to detect the expression of Tim-3 in CD4^+^ T cells and Tregs (Figure 1B).

The proportion of decidual Tregs^Tim-3+^ was highest in the first trimester and subsequently decreased in the second and third trimesters, exhibiting a statistically significant difference, though the proportion of decidual Tim-3^+^CD4^+^ T cells did not show significant differences among different trimesters (Figure 1C). Furthermore, in the first trimester, the proportions of Tim-3^+^CD4^+^ T cells and Tregs^Tim-3+^ in the decidua were significantly higher than in the periphery (Figure 1D). These findings indicated that the presence of Tregs^Tim-3+^ was mainly limited to the first trimester decidua and not in the peripheral blood.

To determine the differences in gene transcription between peripheral and decidual Tregs, we searched for transcriptome-sequencing data from published databases (Figure 1, E and F) (20). PCA revealed similarities between decidua parietalis Tregs (IT) and Tregs in decidua basalis (PT) but showed significant differences with Tregs in periphery (BT) (Figure 1E). Compared with the BT group, the IT group and PT group exhibited common upregulation of genes including *TXNIP*, *LEF1*, *PRG4*, *Linc01089*, *CDC25B*, *SELL*, and *GPA33* and downregulation of genes including *FOSB*, *JUN*, *GZMA*, *CSRNP1*, *DUSP2*, *TNFAIP3*, *CCL4*, *ZFP36*, and *PPP1R15A* (Figure 1F). Next, we evaluated the differential gene expression between PT and BT and observed that the immune-related genes including *IL-32*, *IRF1*, *CD44*, *CD99*, *GZMA*, *TNFAIP3*, and *TNFRSF18*, as well as the transcription factors including *JUN*, *JUNB*, *PRDM1*, *KLF6*, *SOX11*, and *EIF1*, were highly expressed in the PT group, demonstrating that decidual Tregs may be a highly immunosuppressive and differentiated Treg subgroup (Figure 1G). In particular, compared with BT and IT, Blimp-1, encoded by the PRDM1 gene, was highly expressed in the PT group (Supplemental Figure 1; supplemental material available online with this article; https://doi.org/10.1172/jci.insight.179233DS1).

### Tim-3 expression is positively correlated with Blimp-1 in decidual Tregs.

**Figure 2:**
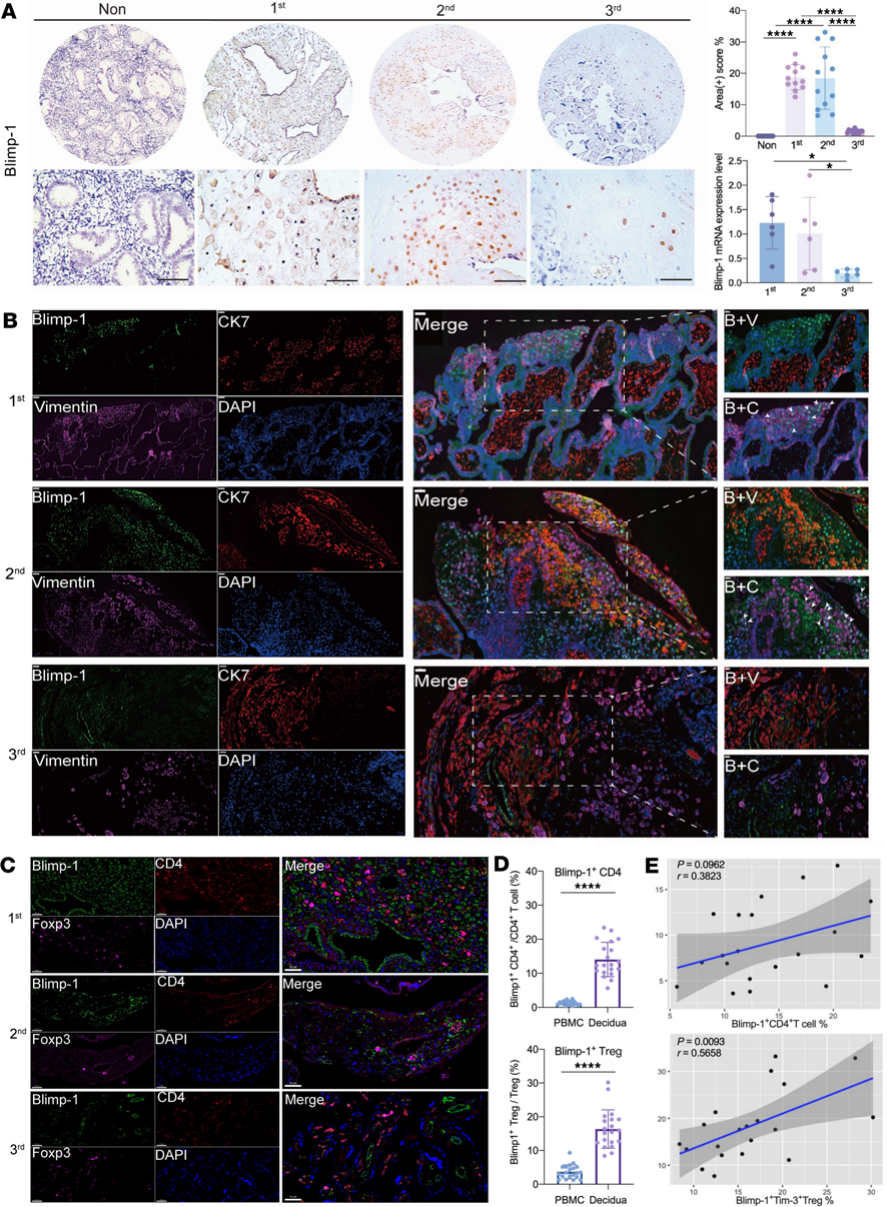
Dynamic expression of Blimp-1 in the first, second, and third trimesters during pregnancy and its correlation with Tim-3. (**A**) Immunohistochemistry (IHC) staining of Blimp-1 expression in different groups; the upper row shows the view under ×100 original magnification, and the lower row shows the corresponding magnified view (×400 original magnification). Scale bar: 100 μm. (**B**) Colocalization of Blimp-1 with CK7 and vimentin in decidua. CK7, a marker of trophoblast cells; vimentin, a marker of decidual stromal cells. The first through third columns are at ×200 original magnification (scale bar: 100 μm), while the fourth column shows the corresponding view under ×400 original magnification (scale bar: 50 μm). (**C**) Colocalization of Blimp-1, CD4, and Foxp3 in decidua Blimp-1^+^ cells and CD4^+^Foxp3^+^Blimp-1^+^ cells, and all images are shown under ×400 original magnification (scale bar: 50 μm). (**D**) Comparison of the proportion of Blimp-1^+^ Tregs and Blimp-1^+^CD4^+^ T cells in PBMCs and decidua of normal early pregnancy by FCM. (**E**) Pearson’s correlation analysis between the proportion of Blimp-1^+^ Tregs and Tim-3^+^ Tregs and between the proportion of Blimp-1^+^CD4^+^ T cells and Tim-3^+^CD4^+^ T cells in decidua. Data are presented as the mean ± SEM, by unpaired, 2-tailed Student’s *t* test (**D**). *P < 0.05, ****P < 0.0001.

To elucidate the relationship between Tim-3 and Blimp-1 in decidual Tregs, we collected human decidual samples at different stages of gestation and detected the expression of Tim-3 and Blimp-1 in Tregs by using immunofluorescence (IF). The results revealed that the expression of Blimp-1 was significantly higher in the decidua of the first and second trimesters than in the nonpregnant endometrium and in the decidua of the third trimester (Figure 2A). Furthermore, to elucidate which cells express Blimp-1, CK7 (a trophoblast marker) and vimentin (a marker of decidual stromal cells), were used to examine the colocalization of Blimp-1, CK7, and vimentin. Blimp-1 was mainly expressed in the extravillous trophoblasts during early pregnancy, whereas its expression decreased during late pregnancy (Figure 2B). Interestingly, certain cells that were neither trophoblasts nor decidual stromal cells expressed Blimp-1 (Figure 2B). Based on the aforementioned RNA-Seq data, we speculated that Blimp-1 is expressed in Tregs.

To further verify whether Blimp-1 was expressed in decidual Tregs, we performed costaining of CD4 (a T cell marker), Foxp3 (a Treg marker), and Blimp-1 using IF. The results revealed that the proportion of Blimp-1^+^CD4^+^Foxp3^+^ Tregs gradually decreased as the pregnancy progressed, with a marked decrease in the second and third trimesters, consistent with the changes observed in Tim-3 expression in the decidua (Figure 2C).

FCM was used to detect the expression levels of Blimp-1 in both the periphery and decidua, which demonstrated a higher proportion of Blimp-1^+^CD4^+^ T cells and Blimp-1^+^ Tregs in the decidua basalis than in the peripheral blood (Figure 2D). The proportions of Tregs^Tim-3+^ and Tregs^Blimp-1+^ were positively correlated; however, no significant correlation between the proportions of Tim-3^+^CD4^+^ T cells and Blimp-1^+^CD4^+^ T cells was observed (Figure 2E). Moreover, the mRNA level of Blimp-1 detected by quantitative PCR (qPCR) was significantly higher in sorted Tim-3^+^ Tregs than that in sorted Tim-3^-^ Tregs (Supplemental Figure 3). Taken together, these results indicate a positive correlation between Blimp-1 and Tim-3 expression in decidual Tregs.

### Blimp-1 directly enhances the expression of Tim-3 in Tregs.

**Figure 3:**
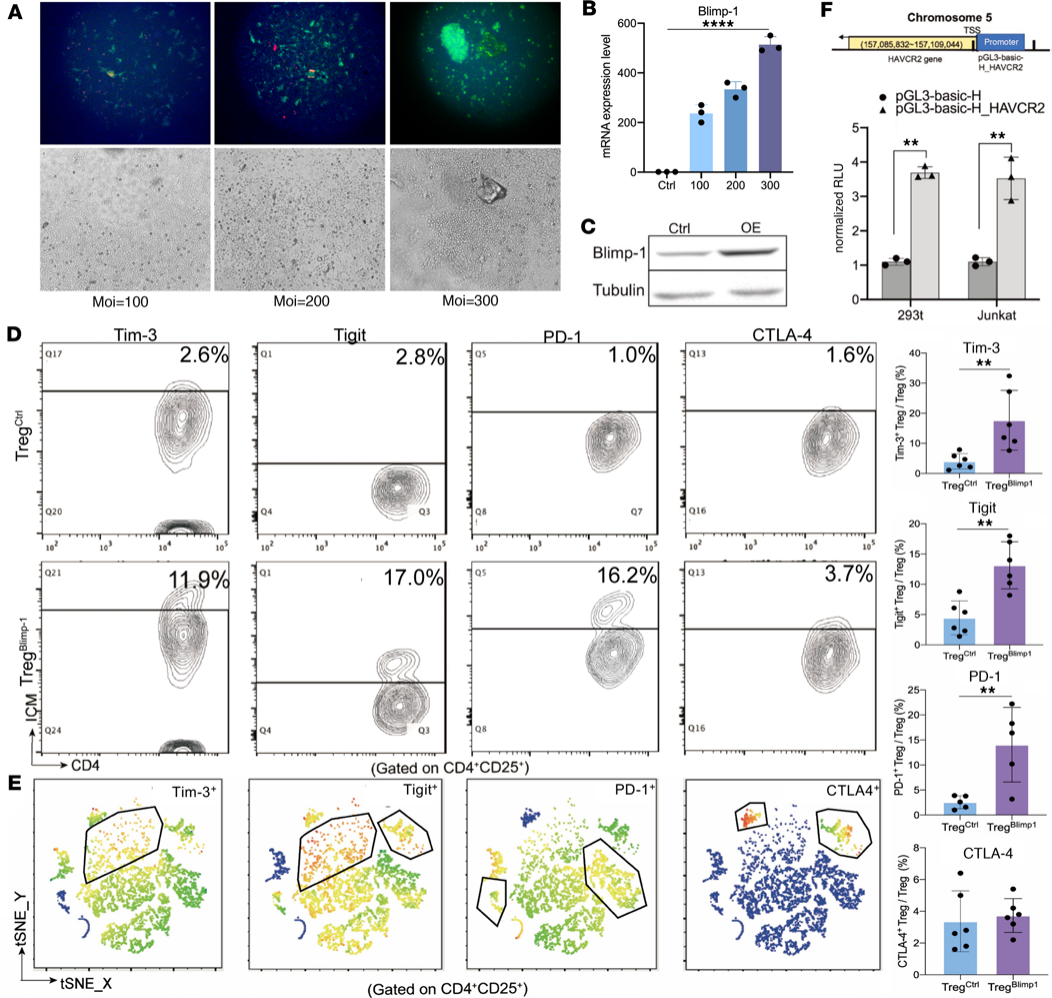
Regulation of Tim-3 expression on Tregs by overexpression of Blimp-1. (**A**) As obtaining primary human Tregs is challenging, for in vitro study, primary CD4^+^CD25^+^ Tregs were isolated from mouse spleens through magnetic-activated cell sorting (MACS). Primary Tregs were transfected with PRDM1-overexpression adeno-associated virus (AVV) when MOI = 100, 200, and 300. ×200 original magnification. (**B**) qPCR analysis of PRDM1 mRNA expression levels. (**C**) Western blotting verified the overexpression of Blimp-1 protein after transduction with AVV in primary Tregs. (**D**) Representative diagrams of FCM and comparison of the expression levels of Tim-3, Tigit, PD-1, and CTLA-4 in Tregs between Treg^Ctrl^ and Treg^Blimp-1^ groups. (**E**) t-Distributed stochastic neighbor embedding distribution plots of Tim-3, Tigit, PD-1, and CTLA-4 (red indicates high expression and blue indicates low expression). (**F**) Cotransfection of Tim-3 promoter luciferase reporter plasmid and PRDM1-overexpression plasmid into HEK293T and Junkat. Comparison between PRDM1+pGL-3_HAVCR2 group and PRDM1+pGL3 empty vector. Data are presented as the mean ± SEM values, by unpaired, 2-tailed Student’s *t* test (**D**). **P < 0.01, ****P < 0.0001.

As obtaining primary human Tregs is challenging, for in vitro study, we used primary CD4^+^CD25^+^ Tregs isolated from mouse spleens through magnetic bead sorting. The CD4^+^CD25^+^ Tregs were transfected with PRDM1-overexpression adeno-associated virus (AVV) at varying MOIs (100, 200, and 300) after CD3/CD28 prestimulation for 24 hours. At an MOI of 300, Blimp-1 gene expression increased more than 300-fold with a significant increase in protein levels (Figure 3, A–C). FCM verified that approximately 50% of the cells were GFP positive (Supplemental Figure 2). These results indicated that transfection at an MOI of 300 yielded the highest overexpression efficiency and could be used in subsequent experiments. To elucidate the role of Blimp-1 in Tregs, the transfected primary Tregs isolated from mouse spleens overexpressing Blimp-1 by AVV were used, and the expression of immune checkpoint molecules (ICMs) was analyzed using FCM (Figure 3D). The results revealed that overexpression of Blimp-1 in Tregs significantly increased the expression of Tim-3, PD-1, and Tigit (Figure 3D). The result of t-SNE analysis showed that Tim-3 and Tigit were coexpressed in Blimp-1–overexpressed Tregs (Figure 3E). Transfection of both the Tim-3 promoter luciferase reporter plasmid and the PRDM1-overexpression plasmid into HEK293T and Junkat cells resulted in a considerable elevation in luciferase reporter gene expression when compared with that in the control group (Figure 3F). This finding suggests that PRDM1 could regulate the expression of Tim-3 at the promoter level.

### Overexpressed Blimp-1 enhances the immune functions of Tregs.

**Figure 4:**
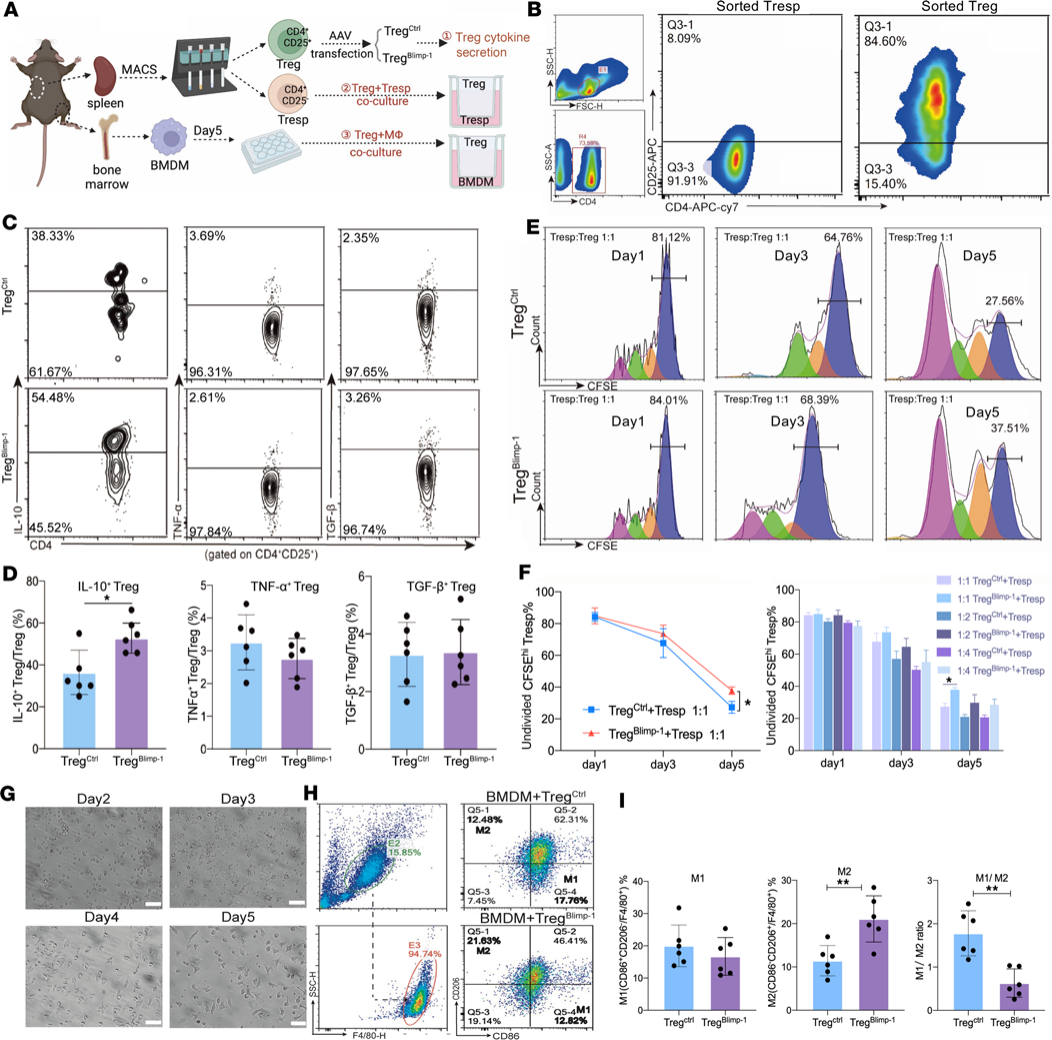
Regulation of Tregs’ immune-suppressive function by overexpression of Blimp-1. (**A**) Diagram illustrating the coculture strategy for investigating the impact of Blimp-1 overexpression on Treg immune function. (**B**) FCM analysis of CD4^+^CD25^–^ Tresp purity after MACS-negative selection and CD4^+^CD25^+^ Treg purity after MACS-positive selection. (**C**) FCM plots for detecting IL-10, TNF-α, and TGF-β secretion in Tregs. (**D**) Bar graph depicting the percentage of IL-10^+^ Tregs, TNF-α^+^ Tregs, and TGF-β^+^ Tregs. (**E**) CFSE proliferation assay of Treg/Tresp = 1:1 coculture on days 1, 3, and 5, with fitted curve of proliferation for Tresps. (**F**) Tregs/Tresps = 1:1, 1:2, 1:4 cocultured for 1, 3, and 5 days; bar graph depicting the proportion of Tresps. (**G**) Representative images of BMDMs cultured for 2–5 days. Scale bar: 100 μm. (**H**) F4/80 purity detection of BMDMs cultured for 5 days. (**I**) Comparison of the percentages of M1 and M2 subsets and M1/M2 ratio after BMDM coculture with Tregs in Treg^Ctrl^ and Treg^Blimp-1^ groups. Data are presented as the mean ± SEM values, by unpaired, 2-tailed Student’s *t* test (**D** and **I**). *P < 0.05, **P < 0.01.

To determine the impact of Blimp-1 overexpression on Tregs’ functions, an experimental design was used, as illustrated in Figure 4A. CD4^+^CD25^–^ responder T cells (Tresps) and CD4^+^CD25^+^ Tregs were isolated from mouse spleens, and their purity was confirmed after MACS (Figure 4B). To evaluate cytokine expression in Tregs, FCM was used to measure the levels of IL-10, TNF-α, and TGF-β in Tregs (Figure 4C). The results showed considerably higher expression of IL-10 in the Treg^Blimp-1^ group than that in Treg^Ctrl^ group; however, no significant difference in the expression of TNF-α and TGF-β was observed between the 2 groups (Figure 4D).

To assess the immunosuppressive function of Tregs^Blimp-1^, the proliferative capacity of Tresps in the coculture system was detected using CFSE (Figure 4E). The results showed that overexpression of Blimp-1 in Tregs inhibited the proliferation of Tresps in a dose-dependent manner, with the most potent impact observed at a Treg/Tresp ratio of 1:1 (Figure 4F). This finding indicated that Blimp-1 overexpression in Tregs enhanced their immunosuppressive functions. Subsequently, we evaluated the impact of Tregs^Blimp-1^ on macrophage polarization by coculturing Tregs^Blimp-1^ with bone marrow–derived macrophages (BMDMs) (Figure 4, G and H). FCM results indicated that Tregs^Blimp-1^ promoted BMDM differentiation toward the M2-like (CD86^–^CD206^+^) subset with a decrease in the M1/M2 ratio (Figure 4I). Moreover, qPCR results showed higher mRNA expression level of arginase 1 (M2 marker) in the Treg^Blimp-1^ group than in the Treg^Ctrl^ group (Supplemental Figure 3).

### IL-27/Blimp-1 axis upregulates the expression of Tim-3 in Tregs.

**Figure 5:**
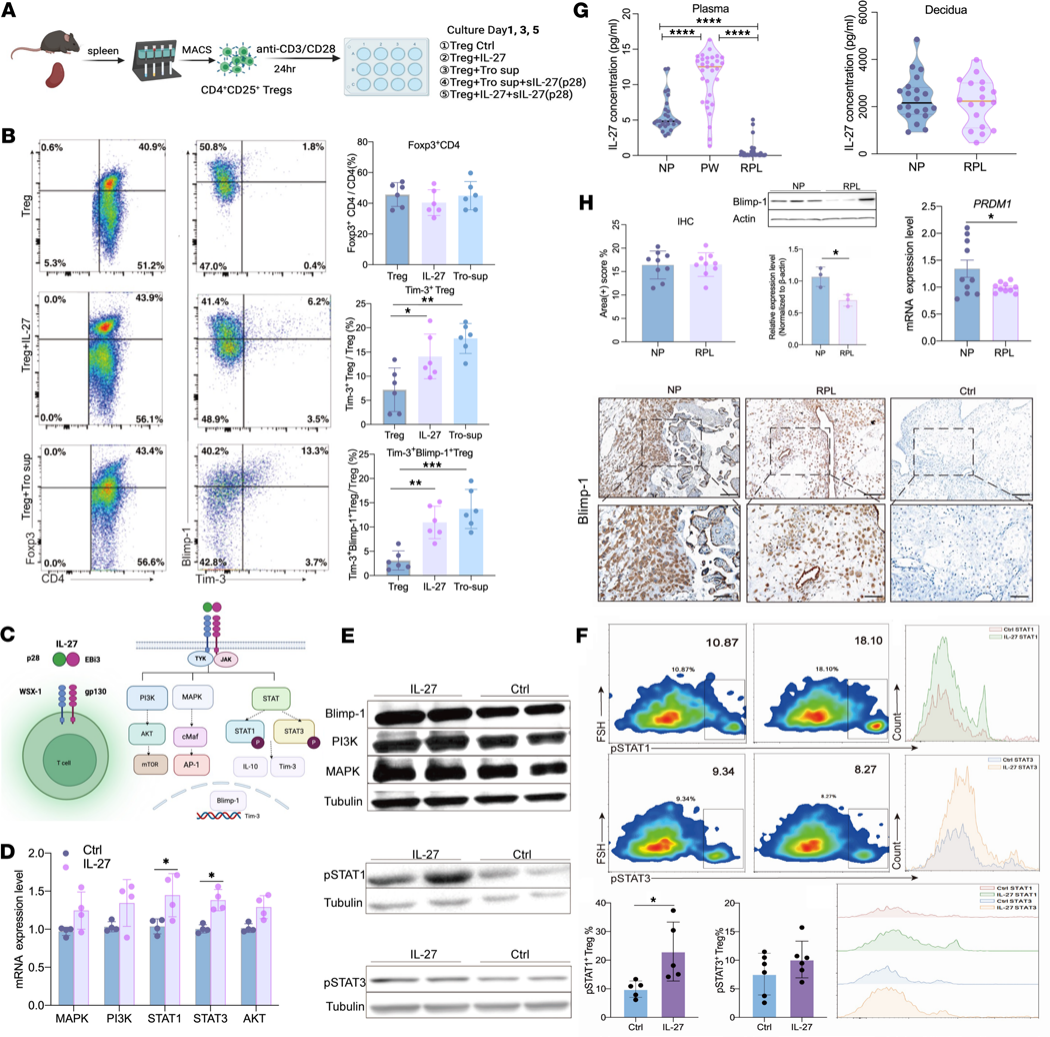
Upregulation of Tim-3 expression on Tregs by IL-27/Blimp-1 axis. (**A**) Schematic representation of in vitro culture strategy divided into 3 groups (*n* = 6): Treg alone (Treg group), Treg + IL-27 (IL-27 group) and Treg + Trophoblast supernatant (Tro-sup group). (**B**) Representative diagram for FCM and comparison of the proportions of Foxp3^+^CD4^+^ T cells, Tim-3^+^ Tregs, and Tim-3^+^Blimp-1^+^ Tregs after 5 days of culture in 3 groups. (**C**) Schematic diagram illustrating IL-27 downstream signaling pathways. (**D**) Detection of mRNA levels of IL-27 downstream signaling molecules, including MAPK, PI3K, STAT1, STAT3, and AKT. (**E**) Detection of the protein levels of IL-27 downstream signaling molecules by Western blotting (WB). (**F**) Detection of phosphorylated Stat1 and phosphorylated Stat3 levels by FCM. (**G**) ELISA measurement of IL-27 levels in plasma and decidua from NP, PW, and RPL groups. NP, normal pregnancy; PW, 1 to 2 years postpartum after a normal delivery; RPL, recurrent pregnancy loss. (**H**) Detection of Blimp-1 expression in decidua by qPCR, WB, and IHC staining in NP and RPL groups. The upper row shows the view under ×200 original magnification (scale bar: 200 μm), and the lower row shows the corresponding magnified view under ×400 original magnification (scale bar: 100 μm). Data are represented as the mean ± SEM via 1-way ANOVA test (**B** and **G**) and unpaired, 2-tailed Student’s *t* test (**D** and **H**). *P < 0.05, **P < 0.01, ***P < 0.001, ****P < 0.0001.

Our previous study has shown that primary trophoblasts can secrete a large amount of IL-27, which induces the differentiation of CD4^+^ T cells into Tregs (3). However, it remains unclear by which mechanism IL-27 regulates the expression of Tim-3 in Tregs. To elucidate this, an in vitro study was performed by adding either exogenous IL-27 or trophoblast culture supernatants to the Treg culture system (Figure 5A). The results showed that both IL-27 and trophoblast culture supernatants promoted the expression of Tim-3 in Tregs, and Tim-3 was mainly expressed in Blimp-1^+^ Tregs (Figure 5B). Additionally, exogenous IL-27 was used and downstream signaling molecules (PI3K, MAPK, STAT1, and STAT3) were detected (Figure 5C and Supplemental Figure 4). The results showed that IL-27 activated the downstream STAT1 signaling, resulting in increased Blimp-1^+^ Tregs and Tim-3 expression (Figure 5, D–F). In contrast, the expression levels of the MAPK and PI3K proteins showed no considerable changes.

To determine whether alterations in the expression levels of IL-27 may play a role in RPL, we investigated whether abnormal expression of IL-27 could be detected by ELISA in the peripheral blood and the decidua of women with normal pregnancy (NP) and RPL (Figure 5G). Our results showed that the IL-27 levels in the peripheral blood of women with RPL were significantly lower than those in women with NP and PW. Despite a decreasing trend in the decidua of women with RPL compared with those with NP, the difference was not statistically significant (Figure 5G). The IHC results showed that compared with the NP group, the proportion of Blimp-1^+^ cells in the decidua tissue of the RPL group was significantly reduced (*P* < 0.05) (Figure 5H).

### Optimal abortion-prone mouse model established through intraperitoneal LPS injection.

To determine the role of the IL-27/Blimp-1 axis in the materno-fetal immune tolerance via the regulation of Tregs, we established an abortion-prone mouse model by injecting LPS intraperitoneally (i.p.) (Supplemental Figure 5). LPS activates intracellular NF-κB signaling by binding to TLR4 on the cell surface and recruits inflammatory cells, leading to Th1 responses (21–23). Compared with the normal control, the LPS groups in low (1.0 μg each), mid (2.5 μg each), and high (5.0 μg each) dosages all showed varying degrees of embryo loss, with the high-dose group exhibiting the highest embryo absorption rate (Supplemental Figure 5). More inflammatory damage to the heart, liver, and kidney was observed in the high-dose LPS group than those in the low- and mid-dose groups (Supplemental Figure 6). The labyrinth (Lab) and junctional zone (Jz) areas were significantly reduced, whereas the Lab/Jz value was significantly increased in the mid-dose LPS group (Supplemental Figure 7). Most blastocysts and placenta in the high-dose LPS were completely absorbed, making morphological evaluation impossible. The qPCR analysis revealed that the placenta of the high-dose LPS group had decreased mRNA levels of the antiinflammatory cytokine TGF-β and increased mRNA levels of pro-inflammatory cytokines including IL-1 and TNF-α (Supplemental Figure 7). Consequently, a mid-dose of LPS (2.5 μg/each) was selected as the optimal dose for subsequent experiments.

### Adoptive transfer of Tregs^Blimp-1^ rescues the pregnancy outcomes in the LPS -induced abortion-prone mouse model.

**Figure 6:**
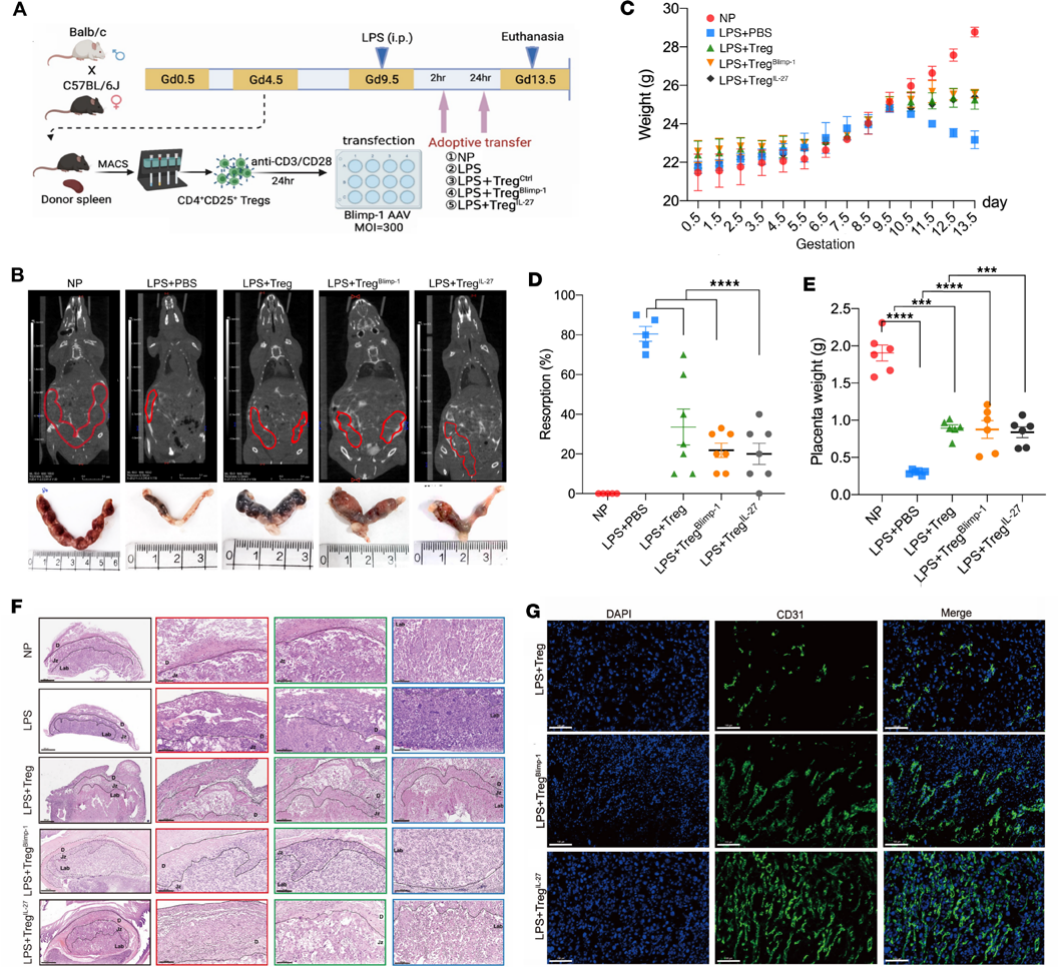
Adoptive transfer of Tregs^Blimp-1^ improves pregnancy outcome in LPS-induced abortion-prone mouse model. (**A**) AT model diagram: LPS (2.5 μg) was injected i.p. on GD 9.5. After 2 hours and 24 hours, 2 × 10^5^ cells/100 μL were transferred via i.v. Tregs were isolated from the spleen of donor mice through MACS. Mice were divided into 5 groups as follows (*n* = 8): NP, LPS, LPS+Treg^Ctrl^, LPS+Treg^Blimp-1^, and LPS+Treg^IL-27^. GD, gestational day. (**B**) Representative images of blastocysts and μCT scan results in different adoptive transfer (AT) groups. (**C**) Dynamic changes in body weight from GD 0.5 to 13.5 in different AT groups. (**D**) Embryonic resorption rates in different AT groups. (**E**) Placenta weight in different AT groups. (**F**) HE staining showing the morphological changes in mouse placenta following AT therapy. The first column shows the view under ×50 original magnification (scale bar: 500 μm), and the second through fourth columns show the corresponding magnified view under ×200 original magnification (scale bar: 200 μm). (**G**) CD31 staining on placental vascular formation in different AT groups. All images are shown under ×400 original magnification (scale bar: 100 μm). Data are represented as the mean ± SEM via 1-way ANOVA test and Tukey’s multiple comparisons test between each 2 groups (**D** and **E**). ***P < 0.001, ****P < 0.0001.

To determine whether Tregs^Blimp-1^ can provide a protective effect against the inflammatory response induced by LPS, we performed adoptive transfer of Tregs, Tregs^Blimp-1^, and Tregs^IL-27^ (Figure 6A). μCT results showed that the LPS + Treg group had dark brown embryos, displaying signs of bleeding or necrosis, whereas the LPS + Treg^Blimp-1^ and LPS + Treg^IL-27^ groups had relatively more complete red embryos (Figure 6B). Compared with the LPS group, groups receiving the transfer of LPS + Treg, LPS + Treg^Blimp-1^, and LPS + Treg^IL-27^ exhibited a considerable reduction in the rate of embryo absorption (Figure 6D). Additionally, a marked decrease in maternal weight was observed after LPS administration on GD 9.5 (Figure 6C). The placental weight in the LPS group was significantly decreased compared with the NP group; however, the placental weight in the transfer group was higher than that in the LPS group (Figure 6E).

Hematoxylin and eosin (HE) staining was used to assess the impact of AT therapy on the mouse placenta (Figure 6F). Compared with the LPS group, the AT therapy groups showed varying degrees of alleviation of pathological damage. Notably, the LPS + Treg^Blimp-1^ and LPS + Treg^IL-27^ groups showed improved placental development and reduced bleeding and necrosis, indicating better morphological recovery than that in the LPS + Treg^Ctrl^ group (Figure 6F). IF staining revealed a limited number of CD31-positive cells in the LPS + Treg group, indicating abnormal vascular formation and inadequate spiral artery remodeling (Figure 6G). In contrast, the LPS + Treg^IL-27^ and LPS + Treg^Blimp-1^ groups exhibited increased numbers of CD31-positive cells, indicating improved vascular development and a more complete vascular morphology (Figure 6G).

**Figure 7:**
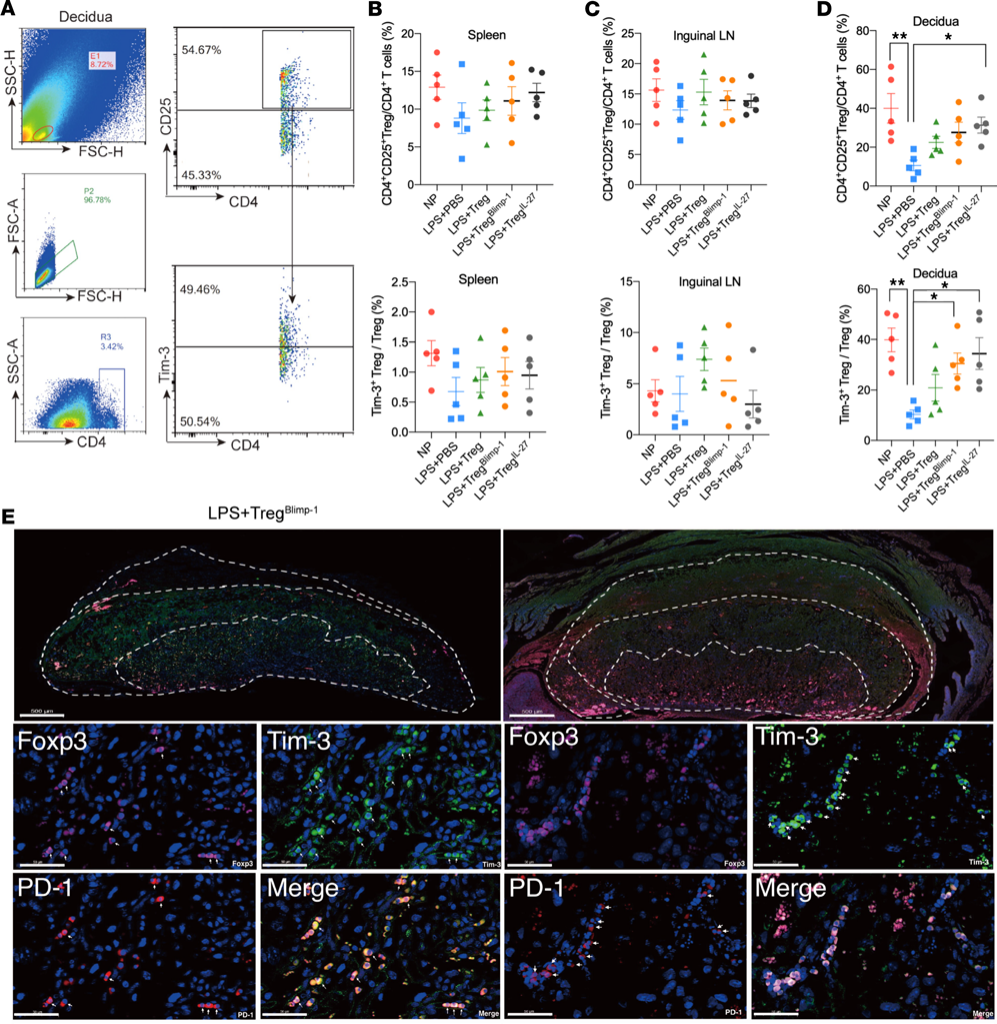
Impact of Treg^Blimp-1^ transfer on mouse pregnancy outcomes. (**A**) FCM gating strategy from decidual tissues. (**B**) Proportions of Tregs and Tim-3^+^ Tregs in the spleen of each AT group were detected by FCM at GD 13.5 (*n* = 5). (**C**) Proportions of Tregs and Tim-3^+^ Tregs in the inguinal lymph nodes of each AT group were detected by FCM at GD 13.5 (*n* = 5). (**D**) Proportions of Tregs and Tim-3^+^ Tregs in the decidua of each AT group were detected by FCM at GD 13.5 (*n* = 5). (**E**) Coexpression of PD-1, Tim-3, CD4, and Foxp3 in the materno-fetal interface was detected using mIHC. The first row shows the placental overall structure in LPS+Treg^Blimp-1^ and LPS+Treg^IL-27^ groups under ×50 original magnification (scale bar: 500 μm). The bottom 2 rows show the placenta in view of ×1,000 original magnification (scale bar: 50 μm). Data are represented as the mean ± SEM via 1-way ANOVA test (**B**–**D**) and Tukey’s multiple comparisons test between each 2 groups. *P < 0.05, **P < 0.01.

The gating strategy of FCM for detecting the proportions of Tregs and Tregs^Tim-3+^ in the decidua is shown in Figure 7A and for the spleen and inguinal lymph node (LN) in Supplemental Figure 8. There were no significant differences in the proportions of Tregs and Tregs^Tim-3+^ in the spleen and the inguinal LN among all the groups (Figure 7, B and C). However, a significant increase in the proportion of Tregs^Tim-3+^ was observed in the decidua of LPS + Treg^Blimp-1^ and LPS + Treg^IL-27^ groups compared with the LPS group (Figure 7D). Multiplex immunohistochemistry (mIHC) results showed that Tim-3, PD-1, and Foxp3 were colocalized in the Lab layer of the placenta in both the LPS + Treg^Blimp-1^ group and the LPS + Treg^IL-27^ group (Figure 7E). These findings indicate that the AT of Treg^Blimp-1^ and Treg^IL-27^ may improve pregnancy outcomes by promoting the placental development and enhancing the materno-fetal immune tolerance, which is characterized by increased Tregs^Tim-3+^ and the expression of negative regulatory molecules in Tregs.

## Discussion

**Figure 8:**
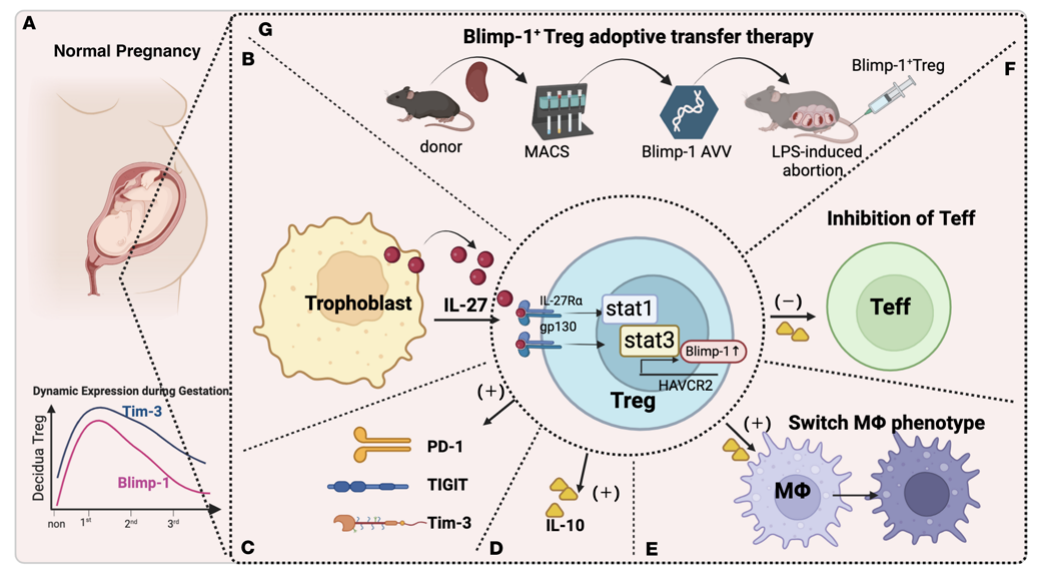
Schematic diagram illustrating the mechanism and intervention strategies of the IL-27/Blimp-1 axis in regulating Tim-3 expression on Tregs. (**A**) Blimp-1 is expressed highest in the first trimester of pregnancy and is closely associated with Tim-3 expression. (**B**) IL-27 increases the expression of Tim-3 and Blimp-1 via the STAT1 signaling pathway. (**C**–**F**) Blimp-1 overexpression promotes the coexpression of Tim-3, Tigit, and PD-1 on Tregs; increases IL-10 secretion; inhibits effector T cell proliferation; and promotes macrophage polarization toward M2 type. (**G**) Transfer of Tregs^Blimp-1^ improves pregnancy outcomes in an abortion-prone mouse model.

We reported the dynamic expression changes of Blimp-1 at the materno-fetal interface during pregnancy, with the highest expression in early pregnancy and a positive correlation with Tim-3 expression (Figure 8A). We further demonstrated that IL-27, acting as the upstream regulator of Blimp-1, could regulate the expression of Tim-3 and Blimp-1 in Tregs mainly via the STAT1 signaling pathway (Figure 8B).

Previous studies on Blimp-1 during pregnancy have mainly focused on its regulatory roles in the early embryo/placental development and decidualization in mice. However, its immunological role at the materno-fetal interface remains unclear. Therefore, this study described the role of Blimp-1 in the regulation of immune cell differentiation and function.

Overexpression of Blimp-1 stimulates the coexpression of ICMs such as Tim-3, Tigit, and PD-1 in Tregs, which enhances their immune suppression or regulatory function. This is accompanied by increased secretion of the immune-regulatory cytokine IL-10, inhibition of effector T cell proliferation, and promotion of macrophage polarization toward the M2-like subset (Figure 8, C–F). The transfer of Treg^Blimp-1^ cells and IL-27 pretreated Tregs provides protection against the pro-inflammatory signals induced by LPS and prevents LPS-induced abortion (Figure 8G).

Blimp-1 is a potential regulatory factor involved in the early embryonic development and the fate determination of primordial germ cells (12, 24). In nonpregnant mouse uteri, the expression of Blimp-1 was extremely low or almost undetectable. Prior to embryo implantation (GD 3.5), low expression was observed in the stromal cells near the uterine lumen epithelium. During the early stages of mouse pregnancy and implantation (GD 3.5 to 6.5), Blimp-1 was significantly upregulated in the primary decidual zone (PDZ), participating in the decidualization process during embryo implantation (13). By using the progesterone receptor-Cre, selective ablation of Blimp-1 in the maternal uterus suppressed the decidualization response, resulting in impaired PDZ formation, ultimately leading to embryonic demise (13). Furthermore, a PRDM1-positive cell subpopulation was detected in human umbilical cord blood, indicating that Blimp-1 may be involved in the late stages of pregnancy (25). Similar expression patterns were observed in our study, which underscores the involvement of Blimp-1 in regulating early embryonic development and decidualization, thereby playing a crucial role in the establishment and maintenance of pregnancy.

In cancer, Blimp-1 regulates the negative costimulatory molecules (CTLA-4, PD-1, Tim-3, and Tigit) to promote T cell exhaustion and aid tumor cells in evading the immune system (18, 26). In acute leukemia, Blimp-1 in CD8^+^ T cells directly binds to the PD-1 promoter, boosting PD-1 expression (19). In our study, the in vitro experiments demonstrated that elevated Blimp-1 levels enhanced Tim-3 expression in Tregs, demonstrating that Blimp-1 facilitates Tim-3 transcription by binding to its promoter region. Zhu et al. (27) observed a similar phenomenon, in which overexpression of Blimp-1 by using a retrovirus in initial CD4^+^ T cells resulted in an increased expression of Tim-3. ChIP-Seq data on tumor-infiltrating CD8^+^ T cells provided evidence of Blimp-1 physically binding to Tim-3 (18). However, owing to limitations in cell availability, our study could not directly confirm Blimp-1 binding to Tim-3 in Tregs using a ChIP-Seq assay. Future research using innovative techniques is warranted to explore the interplay between Blimp-1 and Tim-3 in Tregs.

IL-27 and Blimp-1 are closely linked, and IL-27 can induce Blimp-1 expression to regulate immune responses and inflammation. Heinemann et al. (28) reported that IL-27 and IL-12 collectively counteracted the pro-inflammatory cytokine IL-23 in CD4^+^ T cells by inducing Blimp-1 expression. Chang et al. (29) discovered that IL-27, acting as the upstream molecule of Blimp-1, triggered IL-10 production in Th17 cells via the c-MAF/RORγt/Blimp-1 signaling pathway, thereby promoting the progression of endometriosis. Zhang et al. (30) demonstrated that PRDM1/MAF double-knockout mice were prone to spontaneous colitis and exhibited a phenotype similar to that of IL-10–deficient mice.

IL-27 plays a dual role in promoting inflammation and exerting antiinflammatory effects, making it a key regulatory factor in maintaining a delicate balance between Th1/Th2 and Treg/Th17 responses (31, 32). When functioning as a proinflammatory cytokine, IL-27 recruits more CD4^+^ T cells, stimulates Th1 and T follicular helper cell responses, and hinders Treg stability (33). Conversely, when acting as an antiinflammatory cytokine, IL-27 inhibits CD4^+^ T cell proliferation or migration, suppresses the differentiation and function of Th2 and Th17 cells, and promotes the stability of Tregs. The mechanisms underlying these dual actions are associated with the timing of immune responses, variations in the downstream signaling of IL-27, and the distinct microenvironments it encounters (34). During different stages of pregnancy, the maternal immune system undergoes dynamic changes in response to the varying needs of fetal growth and development (35). Hence, IL-27 may have distinct functions during the different stages of pregnancy (36).

Previous animal studies have demonstrated that IL-10 improves pregnancy outcomes in mice with pregnancy disorders (37–39). IL-10 may be involved in pregnancy maintenance by regulating NK cells, macrophages, and T cells. In addition, the IL-27/IL-27RA signaling pathway was upregulated during decidualization. Absence of IL-27 led to the deficiency of uterine receptivity and fertility in mice (40, 41). Moreover, our previous study found that administrating anti–IL-27 monoclonal antibody in normal pregnant mice did not increase the embryo absorption rates compared to the controls (3). To the best of our knowledge, there is a lack of evidence for the direct injection of IL-27 into abortion-prone pregnant mice. However, supplementing IL-10 in LPS-induced abortion model mice significantly reduces the abortion rate. Maybe IL-27 is also a good option like IL-10, which needs further investigations.

In this study, we detected the levels of IL-27 both in the plasma and in the decidua. Our results indicated that IL-27 levels were markedly reduced in the plasma of women with RPL and not in their decidua. Actually, as our previous study showed (3), IL-27 was mainly secreted by the trophoblasts and was significantly decreased in women with RPL when compared with those in NP. So, what we detected on the levels of IL-27 in the decidua may not adequately reflect the levels of IL-27 in the entire materno-fetal interface microenvironment, including the trophoblasts (41–43). Regarding the interesting results in the level of IL-27 in the plasma, current data demonstrated that IL-27 levels markedly decreased in the women with RPL when compared with those with NP, which might indicate IL-27 could serve as a potential biomarker for RPL. Nevertheless, a larger sample size and prospective cohort study are required for further confirmation.

The AT of immune cells has shown great potential for treating various diseases, including cancer, autoimmune diseases, and chronic inflammation (44, 45). AT therapy involves isolating and expanding specific cell populations, such as T cells, NK cells, Tregs, and DCs, and infusing them into patients to inhibit tumor cells or enhance local immune responses (46). In recent years, Treg-based AT strategies have made considerable progress in treating autoimmune diseases such as type 1 diabetes (T1D), graft-versus-host disease (GVHD), and rheumatoid arthritis (47–50). This strategy involves isolating Tregs from the peripheral blood of patients, followed by ex vivo expansion through stimulation with CD3/CD28 antibodies and IL-2 (51). This leads to the amplification of highly enriched Treg subsets, which are then reinfused into the patient (52). Results from a clinical trial targeting T1D (NCT01210664) showed that 14 adult patients with T1D received ex vivo–expanded autologous CD4^+^CD127^lo/−^CD25^+^ polyclonal Tregs, with a subset of reinfused Tregs exhibiting long-lasting survival (53). Approximately 25% of these cells remained in the circulation after 1 year, supporting the initiation of clinical trials to further validate their therapeutic efficacy (53). Another clinical trial used Tregs from allogeneic hematopoietic stem cell donors in combination with low-dose IL-2 to treat patients with GVHD, establishing a foundation for future adoptive Treg therapies in the posttransplant setting (54).

The etiology of RPL is highly complex, and approximately 50% of cases lack well-defined causative factors (55, 56). Among those women with unknown RPL, approximately 80% have a maternal immune imbalance (57, 58). Although Tregs are mostly used in animal models and preclinical studies, the transfer of Tregs holds promise for treating pregnancy-related disorders, particularly RPL (57). In the CBA/J × DBA/2 model, transferring 2 × 10^5^ fresh Tregs or TGF-β–induced Tregs at E1 increased the proportion of Tregs in the spleen and decidua, elevated the levels of IL-10 and TGF-β in the decidua, and reduced the level of IFN-γ (59). The primary objective of this kind of transfer strategy is to restore self-immune tolerance by increasing the quantity or function of Tregs, thereby reducing the attack of the autoimmune system on the embryo and ultimately preventing or treating spontaneous miscarriages.

Our results demonstrate that IL-27 is a potent regulator of Tregs’ differentiation and can generate Tregs’ capability of immune protection. Using both gene editing via AVV transfection to modify Tregs and IL-27 pretreated Tregs, the rescue effects of the transferred Tregs were further verified in the LPS-induced abortion-prone mouse model. We found that the transferring Blimp-1^+^ Tregs and IL-27–treated Tregs had similar effects in reducing embryo absorption rates and improving the placental vascular development in the LPS-induced abortion-prone mouse model. This could be attributed to the accumulation of Tregs and Tregs^Tim-3+^ in the decidua and the increased expression of negative coinhibitory signaling molecules, including Tim-3 and PD-1, in Tregs, which is beneficial for improving the immune tolerance at the materno-fetal interface. Considering the feasibility issues of gene-edited Tregs in clinical practice, we speculate that exogenously treated Tregs (e.g., using IL-27–pretreated Tregs) may provide similar therapeutic effects. Therefore, our study provides a strategy for the prevention of RPL.

Our study described the exact characteristics and roles of decidual Tregs^Tim-3+^ during early pregnancy. We found that IL-27 promoted the expression of Tim-3 and Blimp-1 in Tregs by upregulating STAT1 signaling. Blimp-1 may bind to the promoter region of Tim-3 to promote its transcription and expression, and Tregs^Tim-3+^ exhibited strong immune tolerance characteristics, including expressing multiple ICMs (Tim-3, Tigit, and PD-1); highly secreting immune-regulatory factor IL-10, inhibiting Tresp proliferation; and promoting macrophage polarizing to M2-like phenotype. The AT of Tregs^Blimp-1+^ and IL-27–pretreated Tregs could improve pregnancy outcomes in an LPS-induced abortion-prone mouse model. Our findings provide insights for further improving the complex network regulation theory of materno-fetal immune tolerance and the development of strategies for the prevention and treatment of pregnancy-related diseases represented by RPL.

This study has some limitations. 1. Although in vitro study indicated that overexpression of Blimp-1 can promote the expression of Tim-3 in Tregs, and luciferase assays revealed that Blimp-1 can enhance Tim-3 transcription by binding to the promoter region, ChIP-Seq technology was not performed to further provide direct evidence of the binding of Blimp-1 to Tim-3 in Tregs. This is because ChIP-Seq technology requires a large number of cells (10^7^~10^8^ cells), which is very difficult to achieve for Tregs isolated from the decidual tissues. In the future, more advanced techniques may be used to further investigate the interaction mechanism between Blimp-1 and Tim-3 in Tregs. 2. Although transfection at an MOI of 300 yielded the highest overexpression efficiency in our study and could be used in subsequent experiments, the efficiency is not so optimal. If using cell lines to be transfected, the transfected efficiency will be better; however, no Treg cell lines could be used. 3. This study found that adoptively transferred Tregs^Blimp-1^ and IL-27–treated Tregs have a certain improvement in the pregnancy outcome of the LPS-induced abortion-prone mouse model. However, tracking and locating the adoptively transferred live cells were not performed; therefore, the proportion of these cells reaching the materno-fetal interface cannot be determined conclusively.

## Methods

### Sex as a biological variable.

Our study exclusively examined female participants and mice because the RPL disease model is only relevant to females.

### Human sample collection.

All participants were recruited from the Department of Obstetrics and Gynecology at the Maternal & Child Health Hospital of Wuhan, China. First-trimester placentas (*n* = 30) (6–9 weeks of gestation) were obtained from voluntary pregnancy termination (termination for nonmedical reasons). Second-trimester placentas (*n* = 15) (12–28 weeks of gestation) were obtained from voluntary abortions after the fetus stopped developing. Third-trimester placentas (*n* = 15) (37–40 weeks of gestation) were obtained from women with normal full-term delivery. Peripheral blood samples were obtained from NP during the first trimester. The detailed information and basic characteristics of the participants are summarized in Supplemental Table 1.

### Isolation of mouse spleen Tregs and AVV transfection.

To prepare the mouse spleen single-cell suspension, we first dislocated and disinfected the mouse, opened the abdomen, and removed the spleen. Afterward, the spleen was ground in a Petri dish with PBS, followed by centrifugation at 500*g* for 5 minutes and lysis of the cells with erythrocyte lysis solution. CD4^+^CD25^+^ Tregs were sorted using a MACS kit (Miltenyi Biotec). Then CD4^+^CD25^+^ Tregs were collected and resuspended in RPMI 1640 complete medium (Cytiva) at 3 × 10^5^ cells/mL. These cells were seeded in a precoated 24-well plate with 1 μg/mL anti-CD3 antibody and incubated overnight. Then anti-CD28 antibody was added to stimulate the cells for 24 hours. For AVV infection, the cells were centrifuged, and the virus solution was added. After gentle mixing, the mixture was left at room temperature for 15 minutes. Subsequently, the cells were cultured, and their infection efficiency was determined by observation under a microscope (TH4-200, Olympus) after 2–3 days.

### Coculture of Tresps or BMDMs with primary Tregs.

In order to isolate BMDMs, femur bone marrow from healthy female C57BL/6 mice was extracted and seeded in complete medium supplemented with M-CSF–conditioned media for 5 days. CD4^+^CD25^–^ Tresps were isolated from mouse spleen by MACS. For coculture of Tregs with Tresps, 1 × 10^5^, 2 × 10^5^, and 4 × 10^5^ CFSE-labeled Tresps were added in the upper chamber, and 1 × 10^5^ Tregs were plated in the lower chamber of a Transwell plate with 0.4 mM membrane. Tresp proliferation was detected by FCM at days 1, 3, and 5, respectively. For coculture of Tregs with BMDMs, BMDMs were added on top of the Transwell chamber. After 48 hours, the cells were harvested; stained with F4/80, CD86, and CD206 antibodies; and analyzed by FCM.

### Western blotting and ELISA.

Tissue or cell samples were lysed in lysis buffer (Beyotime Biotechnology). Samples were separated using SDS-PAGE and transferred to PVDF Transfer Membrane (MilliporeSigma), where specific antibodies were used to visualize the proteins. Relative protein levels were analyzed using the ImageJ software V1.4 (NIH). β-Actin was used as a loading control. Quantification of IL-27 in the plasma and decidua was performed using ELISA (CSB-E16522h, Cusabio), according to the manufacturer’s instructions. All specific antibodies used are listed in Supplemental Table 2.

### IHC and IF.

Paraffin sections of these tissues with a thickness of 5 μm were dewaxed in xylene, rehydrated with graded ethanol, and then washed in PBS. Antigens were repaired by microwaving in 10 mmol/L citrate buffer at a pH = 6.0 (15 minutes). Endoperoxidase activity was quenched using 3% H_2_O_2_ in methanol, and nonspecific sites were blocked with 5% normal serum. The samples were then incubated with primary antibody at 4°C overnight. After washes with PBS 3 times, the sections were overlaid with the secondary antibody and developed in peroxidase substrate solution. The reaction was induced using 3,3′-diaminobenzidine, followed by counterstaining with hematoxylin. Subsequently, the sections were dehydrated through increasing concentrations of ethanol and xylene. Prepared slides were generated from 4 to 6 randomly chosen fields, and images were captured at varying magnifications using an Olympus BX51 microscope along with the Olympus DP70 imaging system. The experimental procedure was replicated 3 times.

### Dual luciferase reporter assay.

The HEK293T cells and Junkat cell lines were both purchased from the China Center for Type Culture Collection in Wuhan, China. HEK293T cells were cultured in DMEM (Gibco) supplemented with 10% fetal calf serum and penicillin-streptomycin and maintained under standard conditions of 37°C and 5% CO_2_. For transfection experiments, cells were seeded at a density of 15,000 cells per well in a 96-well plate and cultured in appropriate medium for 24 hours to achieve 80% confluence. Transfections were conducted using 50 ng of each construct in Opti-MEM (Life Technologies) along with 0.15 μL of HighGene plus transfection reagent (ABclonal RM09014P), followed by a 24-hour incubation period. We transferred 20 μL of the lysis solution into a measurement tube (maintaining consistent sample volume for each instance), then added 100 μL of firefly luciferase assay reagent. We vigorously shook the tube on a shaker 2–3 times to ensure thorough mixing, and after complete homogenization, measured the RLUs. As controls, an empty vector and pRLTK positive control constructs were included in parallel to assess transfection efficiency.

### Quantitative real-time PCR.

Total RNA was isolated using TRIzol reagent (Life Technologies) as per the manufacturer’s protocol. Subsequently, 1 μg of total RNA underwent treatment with gDNA Eraser reagent to mitigate potential genomic DNA interference and was then used for cDNA synthesis, utilizing a kit from Takara Bio. Quantitative real-time PCR amplification was conducted using 2 μL of cDNA in conjunction with Hieff qPCR SYBR Green Master Mix (11198ES03, Yeasen) on a Quantgene q225 instrument (Kubo Technology). Relative mRNA levels were determined using the 2-CT method with normalization to β-actin as the internal control. The primer sequences employed for PCR analysis are provided in Supplemental Table 3.

### Mice and experimental treatments.

Female C57BL/6 mice were mated with BALB/c males (both purchased from the Animal Center of Tongji Medical College), and the presence of a vaginal plug was indicated at GD 0.5. The pregnant mice were randomly divided into 5 groups, with 5–8 mice in each group: 1. NP control group, 2. LPS-induced abortion group, 3. LPS + Treg^Ctrl^ transfer group (AT control Treg), 4. LPS + Treg^Blimp-1^ transfer group (AT Treg overexpressed Blimp-1), and 5. LPS + Treg^IL-27^ transfer group (AT Treg pretreated with IL-27). Embryonic resorption rate, the body weight of pregnant mice, placental vascular development, and peripheral and local Tim-3^+^ Treg phenotype were assessed at GD 13.5.

### Statistics.

All *s*tatistical analysis was performed using GraphPad Prism 8.0. The homogeneity of variances was tested using Levene’s test. Pearson’s correlation analysis method was used to analyze the linear relationship between 2 variables. For the comparison between 2 groups, the *t* test or nonpaired 2-tailed *t* test was used. For the comparison of multiple groups, 1-way ANOVA was used for data with a normal distribution. The data are presented as mean ± SD, and *P* < 0.05 was considered statistically significant.

### Study approval.

All animal procedures were performed in accordance with the approved guidelines of the Institutional Animal Care and Use Committee of Tongji Medical College, Huazhong University of Science and Technology, Wuhan, China [2022] (IACUC Number: 3558). This study was reviewed and approved by the Clinical Trial Ethics Committee of Huazhong University of Science and Technology (Wuhan, China; CTEC number: S154[2021]). Placenta donors gave written informed consent.

### Data availability.

The RNA-Seq data included in this study were sourced from a previously published data set and have been deposited in GitHub (https://github.com/JudithWienke/Human-uterine-Tregs) (20). Raw data for figures presented in this manuscript are available in the Supporting Data Values file.

## Author contributions

SJZ designed and conducted experiments, acquired and analyzed data, interpreted data, and wrote the manuscript. XHH conducted sample collection. XXL performed the IHC experiments and RNA extraction. YJZ helped optimize the IHC studies. XHH, HW, JW, and GSG participated in data interpretation and revision. GM helped in revising the manuscript. AHL contributed to the concept, design, text revision, and final approval. All authors have read and approved the final manuscript.

## Supplementary Material

Supplemental data

Supporting data values

## Version 1 08/22/2024

Electronic publication

## Version 2 08/23/2024

Fixed incomplete affiliation 1.
