# Supplementary material for: IL-27/Blimp-1 axis regulates the differentiation and function of Tim-3+ Tregs during early pregnancy
Source: JCI Insight. 2024 Aug 22;9(16):e179233. doi: 10.1172/jci.insight.179233 (PMC11343602; doi:10.1172/jci.insight.179233)
Supplement: Supplemental data [file jciinsight-9-179233-s279.pdf]

Supplemental Table 1. Clinical characteristics of normal pregnant women in the first (1st), second (2nd) and third (3rd) trimesters of pregnancy.

Clinical characteristics	1st (n=30)	2nd (n=15)	3rd (n=15)	*P*-value
Age（y）	26.61±2.51	27.60 ±7.10	25.46±4.46	> 0.05
BMI（kg/m^2）	20.14 ±3.70	19.58 ±4.82	20.79±6.35	> 0.05
Gestational weeks (w)	8.70 ± 2.10	19.60 ± 3.40	38.2 0± 2.45	< 0.01
Gravidity	1.80 ± 0.85	1.27 ± 0.80	1.40 ± 0.83	> 0.05
Parity	1.573 ±0.82	1.20 ± 0.94	1.33 ± 1.05	> 0.05

Note: Data are presented as the mean ± SEM via one-way ANOVA test.

Supplemental Table 2. The specific antibodies used in the study.

Antibodies	Identifier	Clone	Source
Anti-Stat1 Rabbit mAb	Cat#14994	D1K9Y	Cell Signaling Technology
Anti-Stat3 Mouse mAb	Cat#9139	124H6	Cell Signaling Technology
Anti-Phospho-Stat3 XP Rabbit mAb	Cat#9145	D3A7	Cell Signaling Technology
Anti-Phospho-Stat1Rabbit mAb	Cat#9167	58D6	Cell Signaling Technology
Anti-PRDM1/Blimp1	Cat#ab198287	EPR16655	Abcam
Anti-FITC human CD4	Cat#11-0084-42	GK1.5	Ebioscience
Anti-APC human CD25	Cat#302609	BC96	Biolegend
Anti-Brilliant Violet 421 human CD127	Cat#351309	A019D5	Biolegend
Anti-PE human Foxp3	Cat#12-4776-41	PCH101	Ebioscience
Anti-PE mouse CD366	Cat#134003	B8.2C12	Biolegend
Anti-FITC mouse CD4	Cat#100405	GK1.5	Biolegend
Anti-APC mouse CD25	Cat#17-0251-82	PC61.5	Ebioscience
Anti-ultra-LEAF Purifies mouse IL-27 p28	Cat#516912	MM27-7B1	Biolegend
Anti-mouse monoclonal-β-actin	Cat#66009-1-Ig	2D4H5	Proteintech
Anti-goat anti-mouse IgG (488)	Cat#A23210	—	Babine
Goat ani-rabbit IgG (594)	Cat#A23420	—	Abbkine

Donkey anti-goat IgG (488)	Cat#ab150129	—	Abcam
Donkey anti-mouse IgG (555)	Cat#ab150106	—	Abcam
Goat anti-rabbit HRP	Cat#SA00001-1	—	Proteintech
Rabbit anti-goat HRP	Cat#SA00001-4	—	Proteintech

Supplemental Table 3. The primer sequences used in the study.

Primer	Forward	Reverse
m-TGFβ	TGATACGCCTGAGTGGCTGTCT	TGATACGCCTGAGTGGCTGTCT
m-TNFα	GCCTCTTCTCATTCCTGCTTG	CTGATGAGAGGGAGGCCATT
m-IL-6	CGGCCTTCCCTACTTCACA	CATTTCCACGATTTCCCAGA
m-IL-1β	TGGACCTTCCAGGATGAGGACA	GTTCATCTCGGAGCCTGTAGTG
mIL-10	CGGGAAGACAATAACTGCACCC	CGGTTAGCAGTATGTTGTCCAGC
m-GZMB	CAGGAGAAGACCCAGCAAGTCA	CTCACAGCTCTAGTCCTCTTGG
m-PRDM1	AAGACGTTCGGTCAGCTCTCCA	CTGGCACTCATGTGGCTTCTCT
m-STAT1	GCCTCTCATTGTCACCGAAGAAC	TGGCTGACGTTGGAGATCACCA
m-STAT3	AGGAGTCTAACAACGGCAGCCT	GTGGTACACCTCAGTCTCGAAG
m-AKT	GGACTACTTGCACTCCGAGAAG	CATAGTGGCACCGTCCTTGATC
m-JNK	CGCCTTATGTGGTGACTCGCTA	TCCTGGAAAGAGGATTTTGTGGC
m-Inos	GTTCTCAGCCCAACAATACAAGA	GTGGACGGGTCGATGTCAC
m-Arg1	CCACAGTCTGGCAGTTGGAAG	GGTTGTCAGGGGAGTGTTGATG
m-Actin	TGCGTGACATCAAAGAGAAG	TCCATACCCAAGAAGGAAGG
h-HAVCR2	GACTCTAGCAGACAGTGGGATC	GGTGGTAAGCATCCTTGGAAAGG
h-PRDM1	CAGTTCCTAAGAACGCCAACAGG	GTGCTGGATTCACATAGCGCATC
h-IL-1β	CCACAGACCTTCCAGGAGAATG	GTGCAGTTCAGTGATCGTACAGG
h-TGFβ	TACCTGAACCCGTGTTGCTCTC	GTTGCTGAGGTATCGCCAGGAA
h-STAT1	ATGGCAGTCTGGCGGCTGAATT	CCAAACCAGGCTGGCACAATTG
h-STAT3	CTTTGAGACCGAGGTGTATCACC	GGTCAGCATGTTGTACCACAGG
h-MAPK1	ACACCAACCTCTCGTACATCGG	TGGCAGTAGGTCTGGTGCTCAA
h-GAPDH	GTCTCCTCTGACTTCAACAGCG	ACCACCCTGTTGCTGTAGCCAA

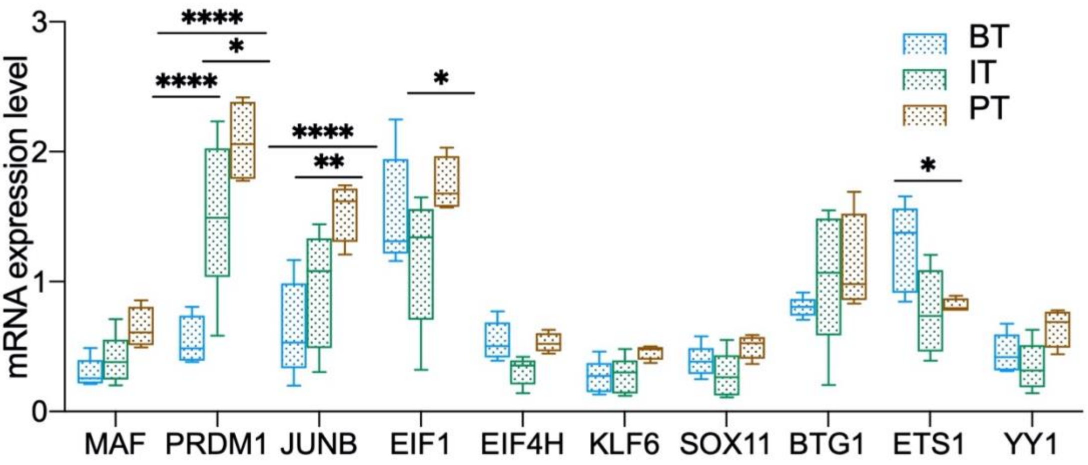

FigS1. Relative expression levels of 10 significantly differentially expressed transcription factors among the BT, IT, and PT groups. BT: peripheral Treg, IT: Treg in decidua basalis; PT: Treg in decidua parietalis. Data are presented as the mean ± SEM via one-way ANOVA test. *P < 0.05, **P < 0.01, ****P < 0.0001.

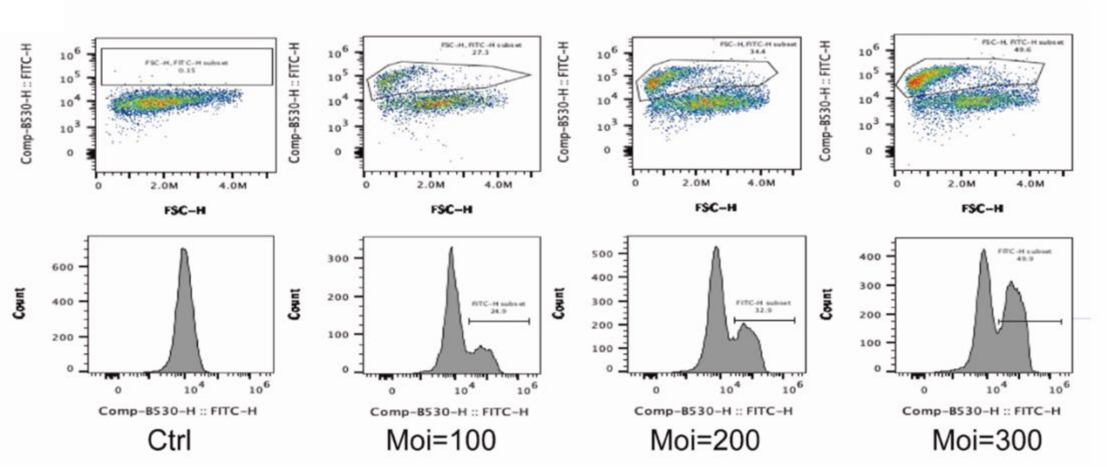

FigS2. FCM analysis of AAV transduction efficiency. At a MOI of 100, the efficiency of AAV in primary Tregs was approximately 20%; At a MOI of 200, the efficiency was approximately 35%; At a MOI of 300, the efficiency could reach 50%.

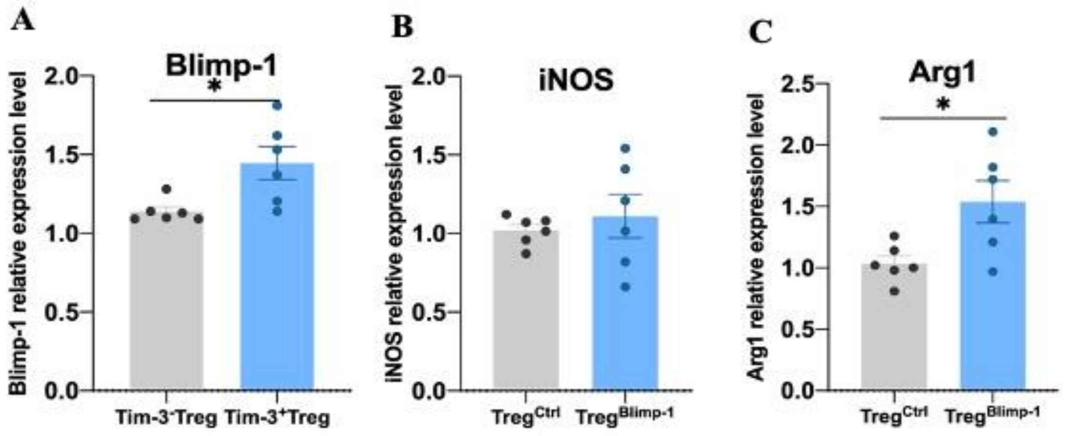

FigS3. Detection for the mRNA expression levels of Blimp-1, iNOS and Arg1 by qPCR. (**A**) Comparison of Blimp-1 mRNA levels in sorted Tim-3^{+} versus Tim-3^{-} Tregs detected by qPCR method. (**B-C**) Comparison of iNOS (inducible nitric oxide synthase; M1 marker) and Arg1 (arginase 1; M2 marker) mRNA levels in BMDM after co-cultured with Tregs only or Blimp-1 overexpressed Tregs detected by qPCR method. Data are presented as the mean ± SEM via unpaired two-tailed Student's *t* test. *$P < 0.05$.

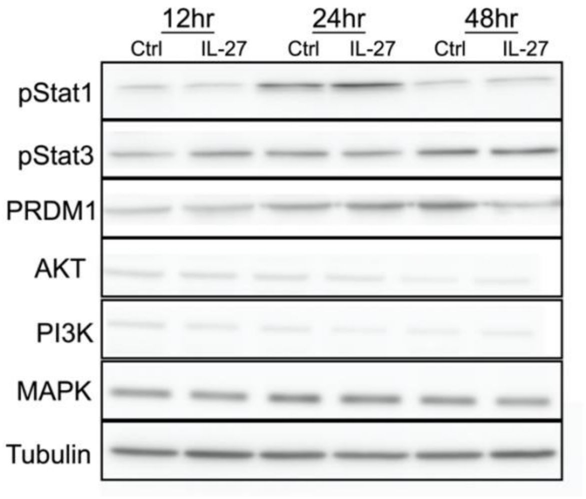

FigS4. The downstream signaling pathway of IL-27. After exogenously adding IL-27 to Junkat cells, WB detection was conducted at 12 h, 24 h, and 48 h, respectively.

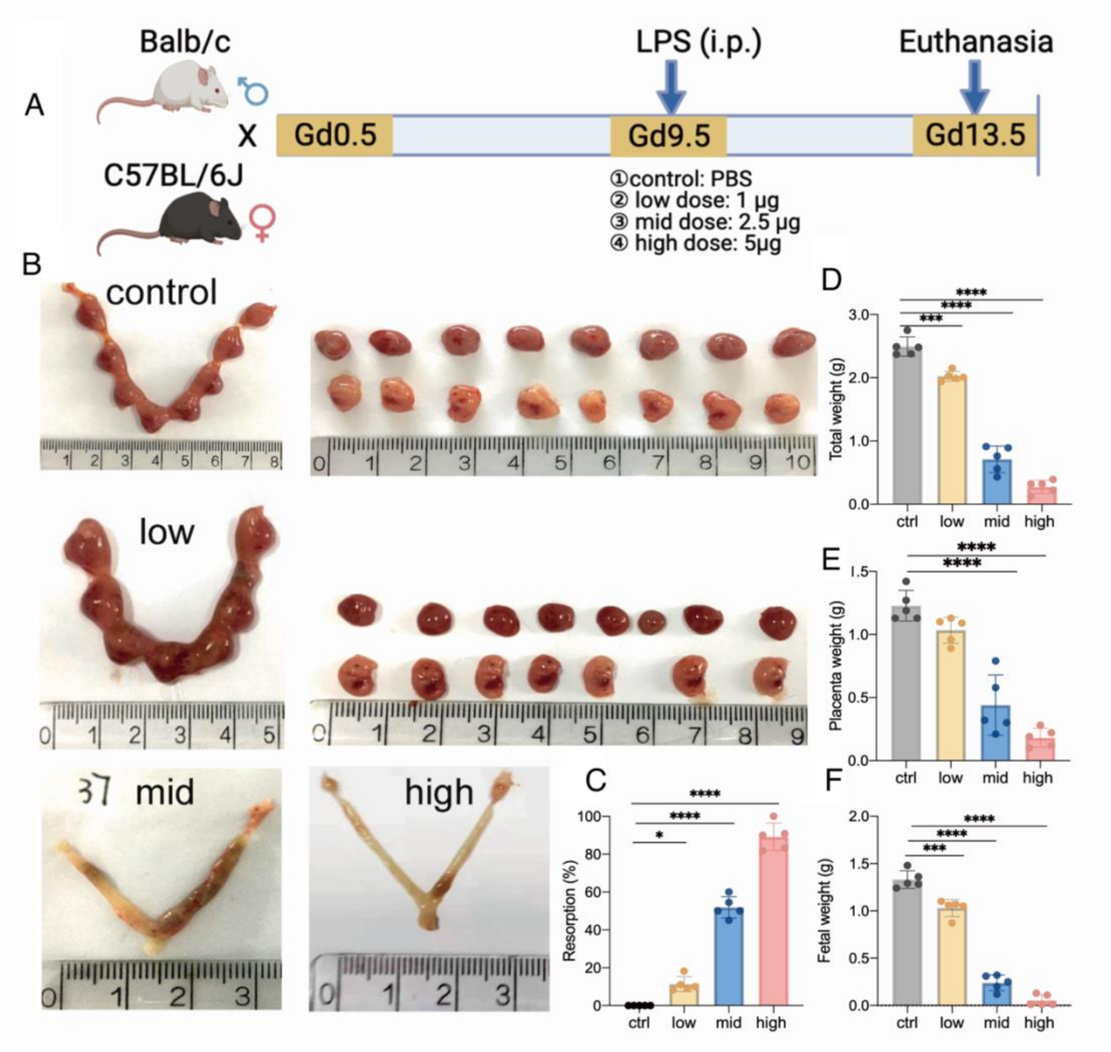

FigS5. Construction of LPS-induced abortion-prone mouse model. (**A**) Establishment of abortion-prone mouse model by using low, mid, and high doses of LPS. **(B)** Pregnancy outcomes in different LPS dose groups. **(C)** Statistical graph of resorption rate in different dosage groups of mice. **(D-F)** Statistical graph of overall weight, placental weight, and fetal weight changes in different dosage groups. Data are represented as the mean ± SEM via one-way ANOVA test. *$P < 0.05$, ***$P < 0.001$, ****$P < 0.0001$.

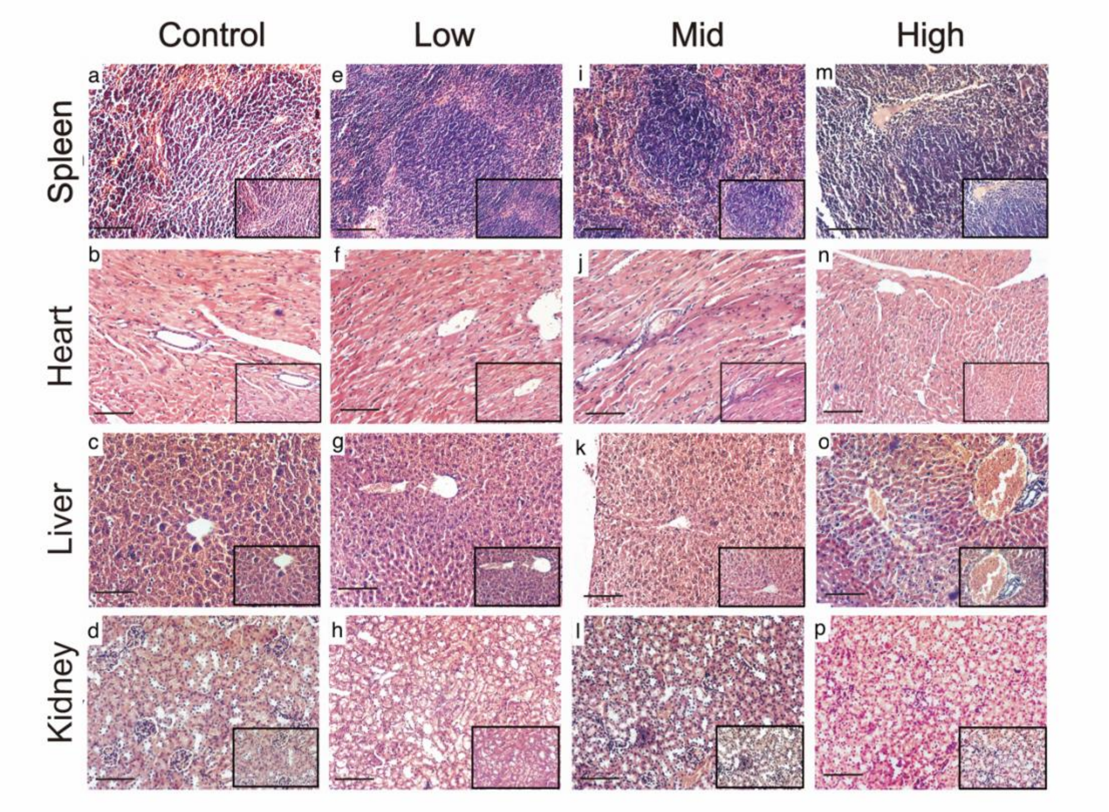

FigS6. HE staining for spleen, heart, liver, and kidney at GD 13.5 in different LPS dose groups of mice. (a-d) PBS control; (e-h) Low-dose LPS group (1 μg LPS / mouse); (i-l) Mid-dose LPS group (2.5 μg LPS / mouse); (m-p) High-dose LPS group (5 μg LPS /mouse); Scale bar: 100 μm.

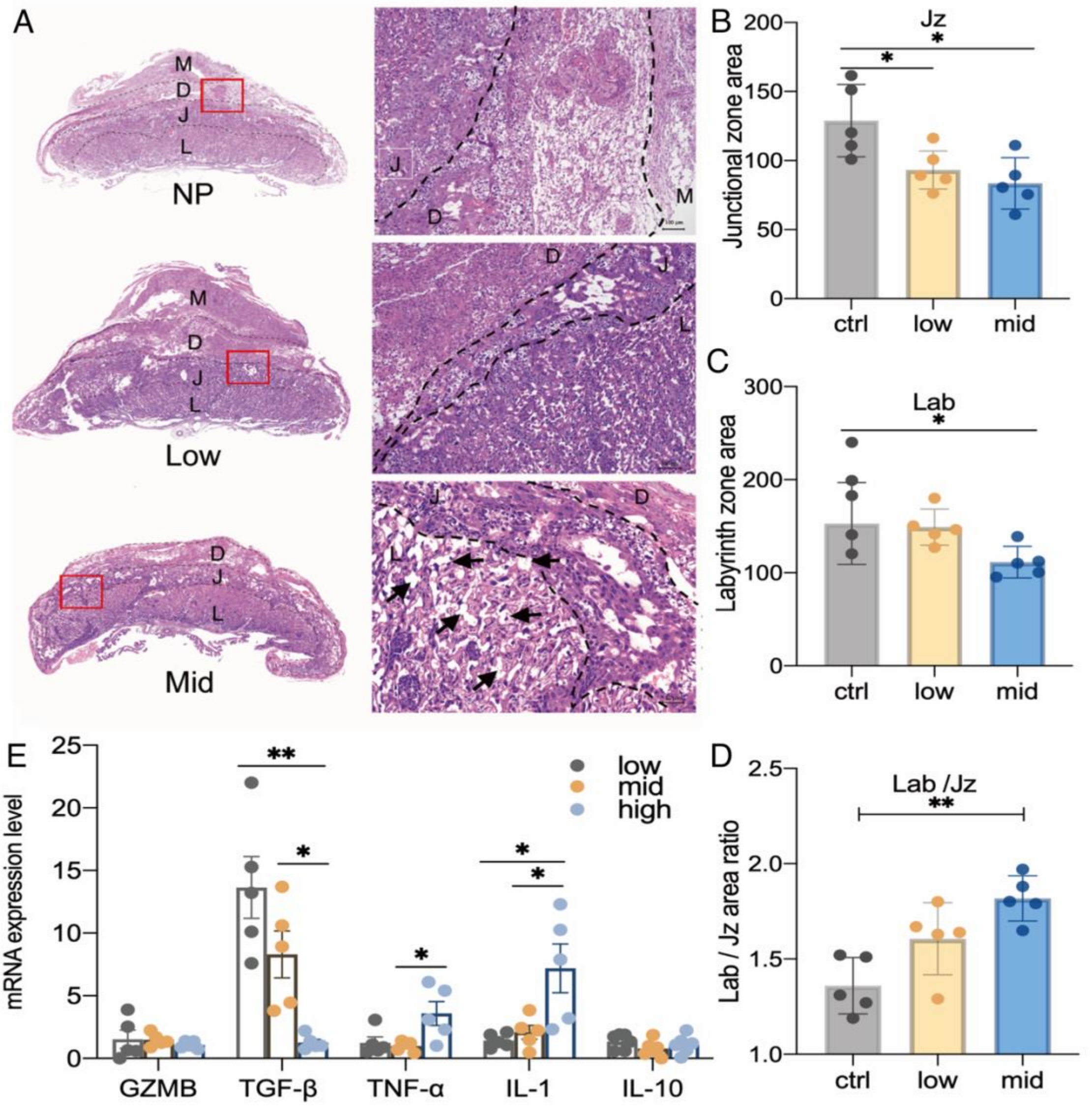

FigS7. Placental morphology and cytokine expression in mouse decidua at GD 13.5. (A) Hematoxylin and eosin staining depicting distinct layers of mouse placenta at GD13.5. (**B-D**) Comparison of the junctional zone (JZ), labyrinth zone (Lab) and Lab/Jz ratio; (**E**) qPCR analysis of cytokine expression levels (GZMB, TGF-β, TNF-α, IL-1, and IL-10 mRNA) in placental tissues of different LPS dose groups. Black arrows indicate cell spaces in the labyrinth zone; Scale: 100 μm. Data are represented as the mean ± SEM via one-way ANOVA test and Tukey's multiple comparisons test between each two groups. *$P < 0.05$, **$P < 0.01$.

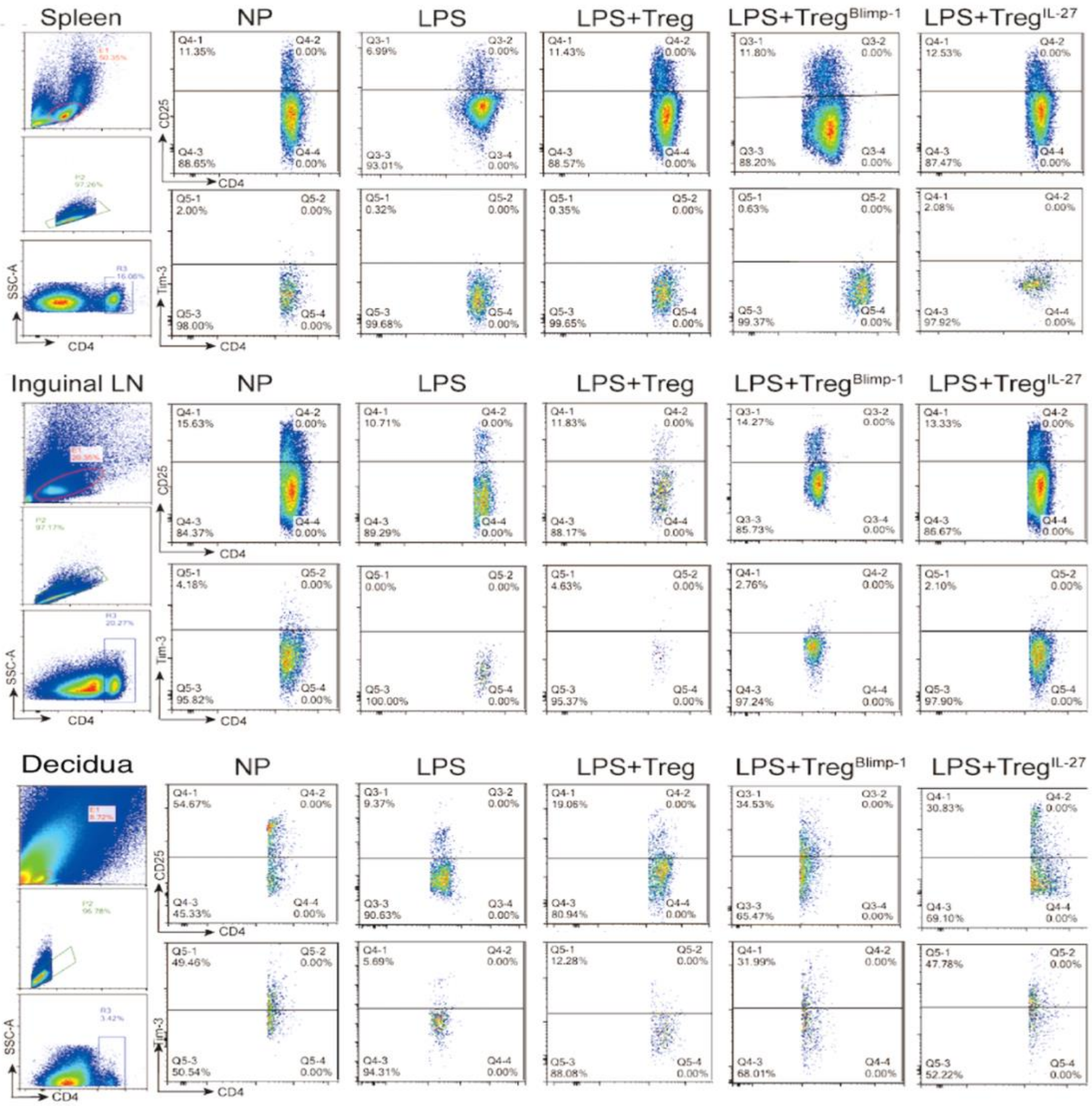

FigS8. Gating strategy and representative figures for detecting the proportions of Tregs and Tim-3^{+} Tregs in the spleen, inguinal lymph nodes and decidua of each group at GD 13.5 by FCM.
